# Prevalence of Back and Neck Pain in Orthopaedic Surgeons in Western New York

**DOI:** 10.5435/JAAOSGlobal-D-21-00252

**Published:** 2022-01-06

**Authors:** Christopher Lucasti, Mark Maraschiello, Josh Slowinski, Joseph Kowalski

**Affiliations:** From UBMD Orthopaedics and Sports Medicine Doctors Buffalo, Buffalo, NY (Dr. Lucasti and Dr. Kowalski), and Jacobs School of Medicine (Mr. Maraschiello and Mr. Slowinski).

## Abstract

**Introduction:**

The physical requirement of orthopaedic surgeons makes them highly vulnerable to musculoskeletal (MSK) injury. Previous studies have shown the prevalence of back and neck pain in orthopaedic surgeons to be approximately 50%. We hypothesize the prevalence of back and neck pain in orthopaedic surgeons in the Western New York region to be similar to what has been previously reported.

**Methods:**

A survey was sent through e-mail to all actively practicing orthopaedic surgeons in Western New York. A total of 94 surgeons were asked to participate, and 53 responded. Data for demographics, back pain, neck pain, and the impact of MSK pain on lifestyle and career practices were collected and compared with previous research.

**Results:**

Seventy-seven percent of respondents reported back pain, whereas 74% reported neck pain, both of which are greater than those seen previously. Sixteen surgeons reported receiving medical treatment currently or in the past for their MSK pain. Fourteen surgeons said that their pain has caused them to adapt their practice and/or operating room setup.

**Conclusion:**

We found the prevalence of back and neck pain in this population to be higher than that previously reported. Additional investigation into the possible causes of the higher prevalence should include the number of arthroscopic procedures done, the amount of time spent wearing lead vests, and the number of hours spent in the operating room by residents.

Orthopaedic surgeons are constantly subjected to physically demanding conditions that put them at an increased risk for developing musculoskeletal disorders (MSDs). Long operating room hours coupled with poor posture and repetitive muscle movements have been previously identified as a source of chronic pain, dysfunction, and decreased quality of life. Previous studies have demonstrated that more than half of all currently practicing orthopaedic surgeons have developed some degree of chronic musculoskeletal (MSK) pain, with cervical and lumbar pain being most frequently reported.^1,2,3,4^ In an attempt to alleviate these work-induced MSDs, a variety of ergonomic interventions have been studied for their efficacy. A clear benefit to these interventions has been established for short-term outcomes, but the long-term effects are less clear.^2,5-7^ These interventions ranged from simple and inexpensive stretches, exercise programs, and breaks to more expensive and cumbersome body suits used during surgery.^5,6,8,9^ Data collection methods for these studies also varied widely, including simple survey data and direct electromyography recordings of postural muscle activation. However, regardless of the intervention or data collection method, the results were similarly beneficial.^5,6,8,9^ These findings support that quick and inexpensive treatments are possible to address the chronic back and neck pain.

Before determining an intervention to alleviate the MSK pain of practicing orthopaedic surgeons, we look to identify the regional prevalence of back pain in the Western New York (WNY) population. The primary goal of this study was to determine whether resident and attending orthopaedic surgeons in WNY have developed work-related back, neck, or other MSK pain.

We hypothesize that at least 50% of practicing orthopaedic surgeons in WNY will report experiencing some level of back, neck, or MSK pain, which would be equal to the prevalence reported in the literature.

## Methods

**Figure 1:**
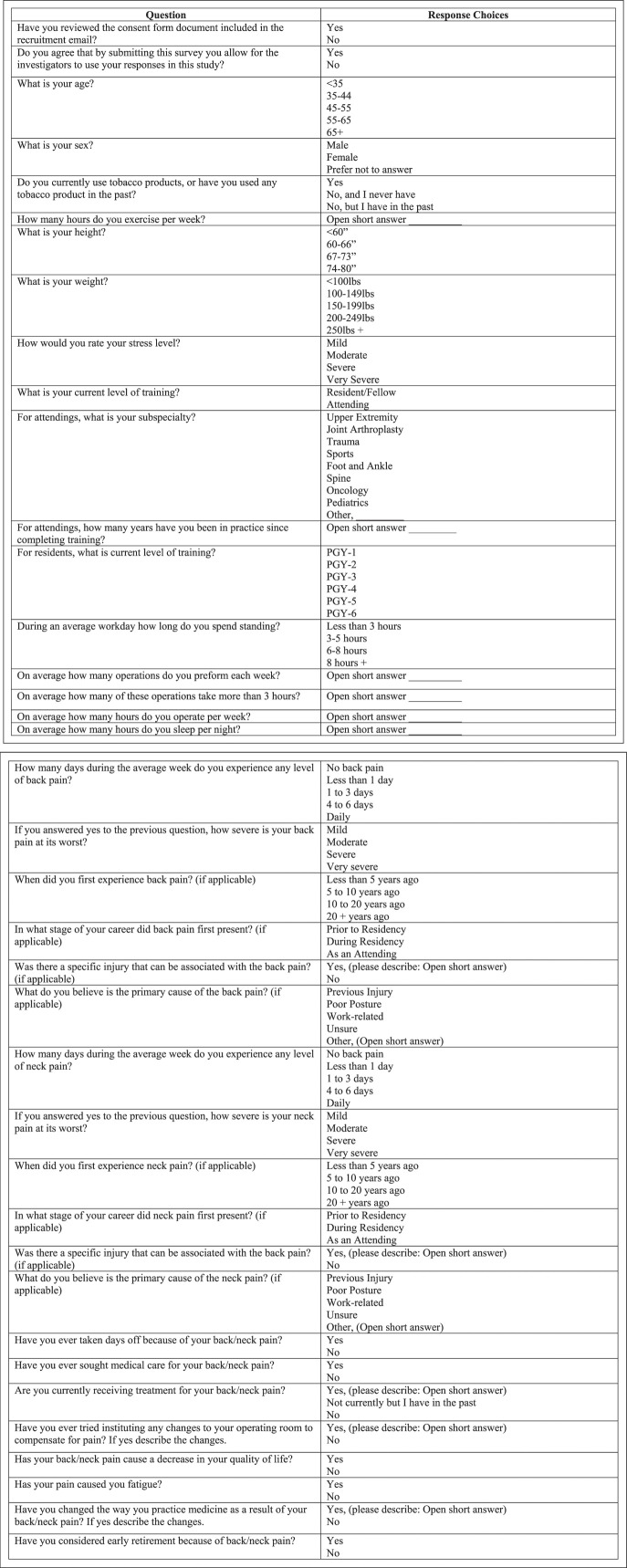
Illustration showing the list of questions and response options that were included in the survey that was sent out to orthopaedic surgeons in Western New York.

We electronically distributed a 38-question survey to actively practicing orthopaedic surgeons (residents, fellows, and attendings) in the WNY area at Excelsior and UBMD orthopaedic groups. See Figure 1 for survey questions. These two groups are private practices and make up most orthopaedic surgeons in the WNY area. The survey disclosed the purpose of the study and asked for consent from each participant to use their data in determining regional prevalence of MSDs in WNY orthopaedic surgeons.

The survey included questions regarding demographic information (age range, sex, smoking history, and height/weight), career information (level of training, specialty, and years in practice), practice schedule (number of days operating each week, number of hours operating each day, and number of hours standing daily), and work-related back, neck, or other MSK pains.

Data from all respondents who chose to complete the survey were deidentified, and the individual results were combined. We then compared the regional data with the prevalence data of previous studies.

## Results

### Demographics

A total of 94 orthopaedic surgeons in WNY were invited to complete the survey, and 53 responded, which corresponds to a response rate of 56%. Of the 53 respondents, 49 were male and 4 were female. Fifteen respondents were younger than 35 years , 17 were between ages 35 and 44 years, 9 were between ages 45 and 54 years, and 12 were between 55 and 65 years . Fifty of the 53 total respondents reported that they have never used any tobacco products, and 3 respondents chose not to answer. Average BMI was found to be 25.7 (SD 3.57) for all respondents. The average number of hours per week spent exercising was 5.2 (SD 3.90) with a range from 0 to 21 hours. The participants averaged 6.5 (SD 0.83) hours of sleep nightly, with 30 respondents reporting less than 7 hours per night.

### Back Pain

When asked about frequency of back pain, 12 respondents reported no back pain at all, 20 reported experiencing back pain 1 or less days a week, 6 reported 2 to 3 days per week, 6 reported 4 to 5 days per week, and 6 reported more than 5 days weekly with back pain. In total, 77% of the respondents reported some level of back pain that affected them on average 2.85 (SD 2.48) days weekly. For the respondents who reported experiencing back pain, 21 reported the pain to be mild, 16 reported moderate pain, and 2 reported severe pain when at its worst. Among the physicians who reported back pain, the onset of back pain was reported to be before residency for 14, during residency for 11, and as an attending for 16. A total of 7 participants reported a history of a back injury that was unrelated to work. When participants were asked what they believed was the most likely source of their back pain, the most common answer was poor posture (12), followed by work (12), unsure (11), exercise/sports (4), and weight gain (1). For only respondents who are current resident surgeons, 67% reported some level of back pain which affected them on average 2.2 (SD 1.73) days weekly. Eighty-two percent of all attending surgeons reported back pain that affected them on average 2.46 (SD 2.62) days weekly.

### Neck Pain

Regarding frequency of neck pain, 14 surgeons reported no history of pain at all. Twenty reported neck pain on average 1 day or less per week, 6 experienced neck pain on 2 to 3 days each week, 6 responded 4 to 5 days per week, and 6 reported neck pain on greater than five days per week. A total of 74% of the respondents reported some degree of neck pain that affected them on average 2.63 (SD 2.33). When asked about the intensity of neck pain at its worst, 21 sufferers rated the pain as mild, 16 rated it as moderate, and 2 as severe. Twelve reported that their neck pain began before residency, 11 during their medical training, and 16 after they became attendings. Only 3 respondents of neck pain reported a history of neck injury that was unrelated to work. When asked what respondents believed to be the primary cause of their neck pain, the most common response was work (15), poor posture (13), unsure (9), and previous injury (2). Eighty percent of the residents reported neck pain that affected them on average 1.73 (SD 1.91) days per week, and 71% of the attending surgeons reported neck pain that affected them on average 2.08 (SD 2.49) days weekly.

### Career and Lifestyle

Of the 53 surgeons who took part in the survey, 15 are current residents/fellows and 38 are attending physicians. The attending surgeons specialize in upper extremity (11), sports (9), joint arthroplasty (6), foot and ankle (3), trauma (3), pediatrics (2), and oncology (2). Self-reported current stress levels were reported as mild in 5 respondents, moderate in 42 respondents, and severe in 6 respondents. For the attending physicians, the average number of years in practice was reported as 14 (SD 9.05) years with a range of 1 to 33 years.

When asked about the duration of a normal work shift spent standing, an average of 6.72 (SD 1.74) hours per day was reported. The average number of operations performed weekly among the 53 respondents was reported to be 12.4 (SD 8.50). The average number of hours spent in the operating room weekly among respondents was 21.2 (SD 10.50) hours, with 6 surgeons reporting 0 to 10 hours, 17 surgeons reporting 11 to 20 hours, 17 surgeons reporting 21 to 30 hours, and 13 surgeons reporting greater than 30 hours in the operating room weekly.

Participants were asked how this pain has affected their professional careers and how they managed their symptoms. Sixteen of 53 respondents reported receiving medical treatment for back or neck pain at some point in their careers, whereas 10 reported actively receiving treatment. Sixteen participants reported that their pain has caused them to reduce the number of hours they operate or modify their operating procedures. Three respondents reported having missed days of work because of chronic back or neck pain, and another participant even reported choosing to stop operating completely because of ongoing back pain. In addition, 8 respondents reported that they have at least contemplated an early retirement because of their chronic pain. Finally, participants were asked how their pain has affected life outside of medicine, and 8 respondents said that it has caused a noticeable decrease in their quality of life. Fourteen participants also reported that their pain caused them to fatigue quicker than they otherwise would have while preforming daily activities.

## Discussion

Orthopaedic surgeons are at a markedly greater risk than the general population for suffering from chronic MSK pain due to the extended hours of preforming surgical procedures in nonergonomic positions.^1,8^ This study assessed the prevalence of back and neck pain and other general MSDs in orthopaedic surgeons practicing in the WNY area. We also explored some general potential causes for and consequences of these symptoms in the careers and lifestyles of the study sample.

Greater than 77% of the respondents reported back pain, whereas 74% reported neck pain. This distribution was consistent among residents/fellows and attending physicians. Similarly, the prevalence of both back and neck pain in these groups was greater than expected based on the reports from previous studies of orthopaedic surgeons in the literature, which determined the prevalence of work-related MSK pain to be between 35% and 60%.^3^ In our survey, respondents with back pain, neck pain, or both most commonly attributed their pain to poor posture, with the second most common reason being work-related injury. The combination of poor posture and work-related injury accounted for 60% of all back pain cases and 72% of all neck pain. These numbers align with previous studies that show approximately two-thirds of MSK pain in surgeons are accounted for by work-related factors.^1^ Causes for the increased incidence in our population may be explained by a variety of factors, including a large proportion of respondents specializing in arthroscopic procedures, trainees spending more time operating than their peers, or random variances within the relatively small sample of participants responding to the survey.

Twenty-four percent of the respondents reported changing their practice to alleviate or accommodate their pain. The reported changes ranged from adjusting their positioning in comparison with the operating table to a single surgeon giving up operating altogether. Previous data showed that between 7% and 18% of the surgeons with MSK pain had to evolve to accommodate their pain. The increased rate seen in this population may be explained by the higher overall prevalence of back and neck pain in our sample.

This study's limitations include the cross-sectional nature of a survey in providing associations without determining causality. The length and details of questions chosen for the survey were also a limiting factor with the goal of maximizing the response rate by minimizing the time required to complete the survey. Response bias is likely because people with MSK pain are more likely to respond to this survey than those who are not sufferers; however, the response rates and prevalence of MSDs in our sample were markedly higher than other related surveys previously performed nationwide. Moreover, no clinical or radiographic evidence was used to determine whether the anecdotal cause of injury was actually medically supported. Our study was inherently subjective in this way based on self-determined medical impressions of our subjects' own conditions. This leaves room for subjective interpretation and additional bias. Finally, this survey was only distributed regionally and encapsulated a relatively small number of total surgeons targeted. The survey had a 56% response rate, far greater than most medical surveys, and the surgeons asked to participate in the survey cover most of the practicing orthopaedic physicians in the WNY area.

Future research should investigate the association between the types of procedures done by surgeons and the development and symptomatology of MSDs. Moreover, studies should investigate what role, if any, lead vests have in causing MSD symptoms because this has been implicated as a cause of back and neck pain in other surgical fields.^10^ Previous research in vascular surgery has shown that 30% of the practicing surgeons experience MSK pain that disrupts their sleep.^11^ Investigating these possible correlations for orthopaedic surgeons will be targeted in future exploration. The reverse situation is also possible because limited hours of sleep may be a factor in causing back and neck pain. Moving forward the primary goal of this long-term study is to determine the prevalence of back pain in a specific regional demographic—orthopaedic surgeons in WNY. With insight from this study, we hope to subsequently test daily stretching and posture routines to determine whether the prevalence and symptoms of back and neck pain in orthopaedic surgeons can be decreased, delayed, or improved in any way through these conservative measures. We hope to use data from this study as a baseline that can be used to compare symptom frequency and severity after the intervention has taken place.
